# Abstract of Meteorological Observations for January, 1855, Made at Philadelphia, Pa.

**Published:** 1855-03

**Authors:** James A. Kirkpatrick


*Abstract of Meteorological Observations for January,* 1855, *made at Philadelphia, Pa. Latitude* 39° 57′ 28″ *N., Longitude* 75° 10′ 40″ *W. from Greenwich.* By PROF. JAMES A. KIRKPATRICK.

| 1855. Jan. | Barometer. Daily Mean | Barometer. Mean Daily Range. | Thermom. Daily Mean | Thermom. Mean Daily Range | Dew Point 2 P. M. | Rel. Humid. 2 P. M. | Rain. and melted Snow. | Prevailing Winds. | Remarks. |
|---|---|---|---|---|---|---|---|---|---|
| | Inches. | Inches. | Deg. | Deg. | Deg. | Hunds. | Inch. | Points. | |
| 1 | 30.297 | .234 | 31.3 | 2.0 | 29.2 | .74 | | NE. | Clear. |
| 2 | 30.484 | .187 | 33.3 | 2.0 | 30.3 | .80 | | NE. | Cloudy. |
| 3 | 30.480 | .052 | 38.3 | 5.0 | 35.3 | .82 | | NE. | Cloudy. |
| 4 | 30.335 | .145 | 44.3 | 6.0 | 43.3 | .85 | 0.054 | NE. | Cloudy; ev. fog; *Rain* during night. |
| 5 | 30.421 | .086 | 40.7 | 5.0 | 33.7 | .67 | | N. | M. and ev. cloudy; aft. clear. |
| 6 | 30.489 | .077 | 41.5 | 3.2 | 37.8 | .96 | 0.092 | NE. | Rain all day. [*highest* 62°. |
| 7 | 30.317 | .171 | 52.3 | 10.8 | 52.7 | .77 | 0.106 | (Var.) | M. fog; aft. cloudy; ev. rain. *Therm.* |
| 8 | 30.586 | .268 | 43.0 | 12.7 | 30.3 | .57 | 0.188 | (Var.) | M. rain; aft. and ev. cl'y; snow during night, about ½ in. *Bar. hig'st* 30.610. |
| 9 | 30.378 | .208 | 36.3 | 4.0 | 33.7 | .89 | | NW. | Cloudy; morning drizzling. |
| 10 | 30.323 | .060 | 33.7 | 4.0 | 24.0 | .44 | | N. | Clear. |
| 11 | 29.957 | .366 | 31.8 | 3.5 | 30.7 | .88 | 0.076 | NE. | Cloudy; morning snow. |
| 12 | 29.745 | .212 | 38.7 | 6.8 | 37.7 | .90 | | SW. | M. fog; cloudy. |
| 13 | 29.517 | .228 | 45.5 | 8.8 | 49.7 | .84 | 0.016 | (Var.) | M. fog; aft. rain; ev. clear. |
| 14 | 30.085 | .568 | 21.3 | 24.2 | 12.7 | .45 | | (Var.) | Clear. |
| 15 | 29.993 | .094 | 29.7 | 8.3 | 28.0 | .69 | | SW. | Cloudy. |
| 16 | 29.946 | .048 | 35.7 | 6.0 | 28.3 | .56 | | (Var.) | M. cloudy; aft. and ev. clear. |
| 17 | 29.905 | .095 | 37.3 | 1.7 | 35.3 | .82 | | NE. | M. and aft. cloudy; ev. fog. |
| 18 | 29.654 | .251 | 39.2 | 3.5 | 29.0 | .51 | | NW. | M. cloudy; aft. and ev. clear. |
| 19 | 29.643 | .050 | 32.7 | 6.5 | 19.7 | .36 | | W.NW. | Clear. |
| 20 | 29.670 | .031 | 32.8 | 2.2 | 25.7 | .61 | | (Var.) | M. and aft. cloudy; ev. clear. |
| 21 | 29.652 | .115 | 36.7 | 3.8 | 33.8 | .96 | 0.529 | NE. | Rain all day, very high wind at night. |
| 22 | 29.540 | .296 | 35.3 | 8.7 | 23.3 | .53 | | W. | M. and aft. cloudy; ev. clear. |
| 23 | 30.015 | .475 | 24.8 | 10.5 | 21.3 | .76 | | W. | Clear. |
| 24 | 29.911 | .104 | 29.5 | 4.7 | 27.3 | .79 | 0.071 | NW. | M. *Snow*, aft. and ev. cloudy. |
| 25 | 29.858 | .054 | 22.7 | 6.8 | 19.0 | .65 | | (Var.) | M. cl'r; aft. & ev. cl'y. *Th. lowest* 16°. |
| 26 | 29.236 | .622 | 29.5 | 7.5 | 27.0 | .84 | 0.300 | (Var.) | Snow during night; day, rain, hail & snow, about 3 in. *Bar. lowest* 29.163 |
| 27 | 29.556 | .320 | 23.3 | 6.2 | 17.8 | .58 | | SW. | Cloudy. [wind during night. |
| 28 | 29.606 | .247 | 36.7 | 13.3 | 31.7 | .65 | 1.169 | NE. | M. cloudy; aft. and ev. rain; high |
| 29 | 29.567 | .258 | 36.5 | 8.5 | 31.0 | .72 | | (Var.) | M. fog; aft. and ev. clear. |
| 30 | 29.904 | .337 | 27.3 | 9.2 | 25.3 | .78 | | SW. | M. and aft. cloudy; ev. clear. |
| 31 | 30.023 | .118 | 25.8 | 3.2 | 25.3 | .78 | | W. | Clear. |
| Means for Jan. 1855 | 29.971 | .206 | 34.4 | 6.7 | 30.0 | .72 | 2.601 | N. 17¾° W., 46–100. | |
| 4 yrs | 29.959 | | 30.7 | | 30.4 | .78 | 2.193 | N. 56° W. 51–100. | |

The Monthly Range of the Barometer was 1.447 inch., and of the Thermometer 46°.

